# Effects of Alginate Oligosaccharide on Testosterone-Induced Benign Prostatic Hyperplasia in Orchiectomized Rats

**DOI:** 10.3390/nu15030682

**Published:** 2023-01-29

**Authors:** You-Jee Jang, Hye-Yeon Jung, Ju-Yeong Myeong, Kwang Hoon Song, Joseph Kwon, Duwoon Kim, Jae-Il Park

**Affiliations:** 1 Department of Biomedical Laboratory Science, Honam University, Gwangju 62399, Republic of Korea; 2 Animal Facility of Aging Science, Korea Basic Science Institute, Gwangju 61751, Republic of Korea; 3 KM Convergence Research Division, Korea Institute of Oriental Medicine, Daejeon 34054, Republic of Korea; 4 Department of BioChemical Analysis, Korea Basic Science Institute, Daejeon 34133, Republic of Korea; 5 BIO3S, Inc., Gwangju 61007, Republic of Korea; 6 Department of Food Science and Technology, Foodborne Virus Research Center, Chonnam National University, Gwangju 61186, Republic of Korea

**Keywords:** alginate oligosaccharide, benign prostatic hyperplasia, 5α reductase type 2, testosterone, dihydrotestosterone

## Abstract

Benign prostatic hyperplasia (BPH) is an age-related disease of the urinary system that affects elderly men. Current treatments for BPH are associated with several adverse effects, thus highlighting the need for alternative agents. Alginate oligosaccharide (AOS), a water-soluble functional oligomer derived from brown algae, inhibits prostate cancer cell proliferation. However, the effects of AOS on BPH and the underlying molecular mechanisms remain unclear. Therefore, here, we aimed to investigate the therapeutic potential of AOS in BPH by using human benign prostatic epithelial cells (BPH-1) and a rat model of testosterone-induced BPH. Treatment with AOS inhibited in vitro and in vivo proliferation of prostatic epithelial cells and the testosterone-induced expression of androgen receptor (AR) and androgen-associated genes, such as those encoding 5α-reductase type 2 and prostate-specific antigen. Oral administration of AOS remarkably reduced the serum levels of dihydrotestosterone (DHT) and testosterone as well as the expression of proliferating cell nuclear antigen, inflammatory cytokines, and enzymes, which showed increased levels in prostatic tissues of rats with testosterone-induced BPH. Taken together, these data demonstrate that AOS suppresses testosterone-induced BPH in rats by downregulating AR and the expression of androgen-associated genes, supporting the hypothesis that AOS might be of potential use for the treatment of BPH.

## 1. Introduction

Benign prostatic hyperplasia (BPH) is defined as the enlargement of prostatic stromal and epithelial cells, which results in a noncancerous increase in the size of the prostate [1]. BPH is one of the most common diseases worldwide, and its risk increases linearly with age, its development beginning at approximately 40 years of age [1,2]. Although BPH itself is not fatal, it is a significant cause of morbidity owing to the development of lower urinary tract symptoms (LUTS). LUTS, such as frequent urination, trouble starting to urinate, weak stream, inability to urinate, nocturia, or loss of bladder control, lead to an increased risk of obstruction of the urethra, acute urinary retention, bladder stones, hematuria, recurrent urinary tract infection, and renal failure [3,4], which ultimately have an overall negative impact on the quality of life.

During aging, enlargement of the prostate gland results in increased proliferation of stromal and epithelial cells of the prostate, and a relationship exists between inflammation of the prostate and BPH development [5,6]. Inflammation, especially chronic inflammation, is an important factor in the pathogenesis of prostatic hyperplasia, which is observed histologically in specimens of BPH [7,8]. In addition, most tissue samples collected from patients with BPH are infiltrated by a vastly increased population of T lymphocytes, which release a variety of inflammatory cytokines, such as interleukin (IL)-1β, IL-2, IL-4, IL-6, interferon (IFN)-γ, and tumor necrosis factor (TNF)-α, which play important roles in the maturation of the stroma and development of stromal nodules in BPH [9].

Besides inflammation, alterations in the levels of major testicular hormones, such as androgens, are also associated with prostate enlargement in BPH [1,2,10,11]. Androgen replacement in patients with hypogonadism leads to prostate enlargement [12]. Patients with BPH treated with the antiandrogen flutamide showed significantly decreased prostate volume within 6 months of treatment [13]. However, antiandrogen therapy is no longer used in therapeutics because of adverse reactions such as serious hepatotoxicity and liver disease [14,15]. Thus, treatment for patients with BPH was replaced with 5α-reductase inhibitors, which are better tolerated and inhibit BPH development more effectively. Finasteride, which specifically inhibits the 5α-reductase type 2 (SRD5A2), suppresses the conversion of testosterone into dihydrotestosterone (DHT) and is more effective in reducing the periurethral area, prostate gland size, and urinary obstructive symptoms in patients with BPH [16,17]. Another 5α-reductase inhibitor, dutasteride, which inhibits both type 2 and 1 5α-reductase, reduces the serum levels of DHT levels more effectively than does finasteride [18]. Therefore, androgens and androgen receptor (AR)-mediated signaling may contribute to an increase in the prostatic volume, and 5α-reductase inhibitors that suppress testosterone conversion into DHT and decrease the DHT-induced expression of prostate-specific antigen (PSA) are effective medicines for reducing prostate enlargement in patients with BPH. However, both finasteride and dutasteride are associated with sexual dysfunction, including erectile dysfunction, decreased libido, decreased ejaculation, headache, gastrointestinal discomfort, and dizziness [19,20]. Therefore, there is an urgent need to identify alternative treatments that have fewer adverse effects.

Numerous plant-based products (phytomedicines) have been increasingly used as alternative or complementary medicines for the treatment of BPH. Herbal-derived products, such as extracts of Saw palmetto (*Serenoa repens*), *Pygeum africanum,* and *Urtica dioica*, as well as active ingredients such as cernitin and β-sitosterol have been tested in clinical trials for the treatment of BPH [21,22,23]. They have been shown to significantly lessen nocturia and frequency of urination and diminish the overall symptomatology of BPH, while causing no significant adverse effects [24,25,26]. Although classic literature and case reports support the clinical efficacy of different natural formulas in alleviating symptoms of BPH, their underlying mechanisms have not been evaluated. Such information is essential for assessing the overall clinical efficacy and safety of natural products, including functional foods.

Alginate oligosaccharide (AOS) is composed of β-D-mannuronic acid (mannuronic acid) and α-L-guluronic acid (guluronic acid) homogeneously or heterogeneously linked through 1→4 glycosidic bonds, forming linear dimers within the larger polymer [26]. AOS shows non-toxic and non-immunogenic characteristics and is a natural material used in a wide variety of pharmaceutical and food products. It is attractive for biomedical applications owing to its beneficial health effects, including postulated anti-obesity [27], antitumor [28], anti-apoptotic [29], anti-inflammatory [30], and antioxidant [31,32] effects. AOS suppresses the growth and proliferation of prostate cancer cells via the Hippo/YAP pathway by inhibiting ST6Gal-1 promoter activity [28]. However, little is known about the usefulness of AOS for the treatment of BPH. Therefore, in this study, we aimed to investigate whether AOS could impact hyperplasia of prostatic stromal and epithelial cells, prostate size, hormone levels, and inflammatory cytokines in BPH.

## 2. Materials and Methods

### 2.1. Reagents and Antibodies

Testosterone propionate (TP, PHR2026) and finasteride (FN, F1293) were purchased from Sigma–Aldrich (Whitehouse Station, NJ, USA). Antibodies against SRD5A2 (sc-293232), AR (sc-7305), PSA (sc-7316), proliferating cell nuclear antigen (PCNA), cyclin D1 (sc-8396), cyclin B1 (sc-7393), inducible nitric oxide synthase (iNOS, sc-7271), cyclooxygenase-2 (COX-2, sc-376861), and glyceraldehyde-3-phosphate dehydrogenase (Gapdh, sc-47724), and horse-radish peroxidase-conjugated secondary antibodies (mouse-HRP, sc-516102; rabbit-HRP, sc-2357) were purchased from Santa Cruz Biotechnology Inc. (Dallas, TX, USA).

### 2.2. AOS Production

AOS was prepared and supplied by BIO3S, Inc. (Gwangju, Republic of Korea). Briefly, sodium alginate (KIMIKA Co., Tokyo, Japan) was depolymerized by alginate lyase from *Corynebacterium* sp. at 30 °C for 72 h and concentrated by ultrafiltration through a polyethersulfone membrane (AquaCell Membrane Tech. Co., Ltd., Wuhan, China) with a molecular weight cut-off of 100 kDa. The concentrated AOS was sterilized, lyophilized by freeze dryer, and stored at 4 °C until use. The prepared AOS was further dissolved in sterilized distilled water at concentrations of 1, 3, 10, or 30 mg/L.

### 2.3. Cell Culture

Human prostate epithelial (BPH-1) cells were cultured and maintained as previously described [33]. Briefly, cells were maintained in RPMI 1640 (Thermo Scientific, Waltham, MA, USA) supplemented with 20% fetal bovine serum (FBS). After 1 or 2 days, BPH-1 cells were further cultured with serum-starved conditions for 16 h. Next, the cells were co-incubated with testosterone propionate (TP, 10 nM), finasteride (FN, 50 μM), and AOS (1, 3, and 10 μg/mL) for 24 h, and then cells were harvested for real-time quantitative PCR (RT-qPCR) and Western blot analysis.

### 2.4. MTT Assay

Cell viability and proliferation were analyzed using a Quick Cell Proliferation Assay Kit (BioVision, Mountain View, CA, USA) according to the manufacturer’s instructions. BPH-1 cells were seeded in 96-well plates (1 × 10^5^ cells/well) and cultured at 37 °C and 5% CO_2_. For the cell viability assay, media containing various concentrations of AOS (0, 0.1–300 μg/mL) was added and incubated for 24 h. For the cell proliferation assay, media containing three different concentrations of AOS (0.1, 1.0, or 10 μg/mL) was added and incubated for 1, 3, and 5 days. After incubation, 20 μL of MTT solution (final concentration 0.5 mg/mL; BioVision) was added into each well before being returned to incubate at 37 °C for 3 h. Then, the medium in each well was removed and replaced with 150 μL of dimethyl sulfoxide (Sigma–Aldrich) to ensure the dissolution of the crystals prior to incubation at room temperature for 15 min. The absorbance was determined at 570 nm using a microplate reader (SpectraFluor, TECAN, Sunrise, Austria).

### 2.5. Animals Experiments

Male Sprague-Dawley rats (6-week-old, weighing 180–200 g) were purchased from Samtako BioKorea (Seoul, Republic of Korea). They were housed in groups on a light/dark cycle (10-h dark, 14-h light) in a 22 °C temperature-controlled room. All animal procedures were performed in accordance with the Institutional Animal Care and Use Committee of the Korea Basic Science Institute, Korea (KBSI-IACUC-22-20).

A rat model of BPH was established as previously described [33]. Briefly, the testes of the rats were removed to prevent the effect of endogenous testosterone, except for the negative control group in the sham-operated group. For BPH studies, the castrated rats were induced by intraperitoneal (i.p.) injection of 5 mg/kg testosterone propionate (TP), for 28 days. These castrated groups were intragastrically administrated daily for 28 days with a vehicle, finasteride (5 mg/kg), or AOS (10 mg/kg; Low, 30 mg/kg; High), respectively. Oral-fed finasteride was used as a positive control group. The rats were weighed weekly during the experiment. At day 29, prostates were collected, weighed, and fixed in 4% paraformaldehyde for histological and immunohistochemical (IHC) analysis. The prostatic index (PI) was calculated using the following formula: PI = gross wet weight of prostate/weight of whole animal × 100%.

### 2.6. Blood Collection and Biochemical Analysis

All rats were fasted overnight. Blood was obtained by cardiac puncture, collected in a serum separating tube (BD Vacutainer^®^ SST™ tubes), and centrifuged at 3500× g for 10 min. The serum was separated and stored at −70 °C until assays were performed. Testosterone levels were analyzed using Samkwang Medical Lab (Seoul, Korea). DHT concentrations were measured using ELISA kits (ALPCO Diagnostics, Salem, NH, USA) according to the manufacturer’s protocol. Values were expressed as ng per mg protein.

### 2.7. Histopathological Analysis

After deparaffinization of prostate tissues, hematoxylin and eosin (H&E) staining and IHC were performed on 5-μm-thick sections. For H&E staining, sections were stained with hematoxylin and eosin. For IHC sections, sections were incubated with primary antibodies (1:500 dilution) and secondary antibodies (1:1000 dilution) and then detected using 3, 3′-diaminobenzidine (DAB). Images were visualized under optical microscopy (BX53, Olympus Corp, Tokyo, Japan), and the thickness of epithelial layers was measured using ImageJ software (National Institute of Health, Bethesda, MD, USA).

### 2.8. RNA Isolation and RT-qPCR Analysis

Total RNA from cultured cells or prostate tissues was extracted using TRIzol reagent (Invitrogen, Carlsbad, CA, USA), and cDNA were generated using reverse-transcribed using the RevertAid M-MuLV reverse transcriptase kit (Fermentas, St. Leon-Rot, Germany), according to the manufacturer’s instructions. RT-qPCR was performed on a Rotor-Gene Q 5plex using the QuantiTect SYBR Green PCR Kit (QIAGEN, Hilden, Germany). Actb was used as an internal control. Primers used for PCR are listed in Table 1.

### 2.9. Western Blot Analysis

Cultured BPH-1 cells or prostatic tissues collected from each group were homogenized using a cold radioimmunoprecipitation assay (RIPA) buffer containing protease and phosphatase inhibitors (Thermo Scientific, Waltham, MA, USA). Protein samples (50 µg each) were subjected to sodium dodecyl sulfate-polyacrylamide gel. After electrophoresis, transferred membranes were incubated with a primary antibody (1:500 final dilution) and secondary IgG (1:1000 final dilution). Signals were detected via enhanced chemiluminescence and visualized using the ChemiDoc Imaging System (Azure Biosystems, Dublin, CA, USA).

### 2.10. Statistical Analysis

All quantitative data are presented as mean ± standard deviation. Statistical analysis was performed with GraphPad Prism 5 software (GraphPad Software, La Jolla, CA, USA). The significance of differences was evaluated using a one-way analysis of variance (ANOVA) followed by Dunnett’s test for comparisons among multiple groups. A significant *p*-value in the figures is reported as the asterisks denote statistical significance (*, *p* < 0.05; **, *p* < 0.01; ***, *p* < 0.001).

## 3. Results

### 3.1. Effect of AOS on the Proliferation and Prostate-Related Gene Expression of BPH-1 Human Prostate Epithelial Cells

**Figure 1:**
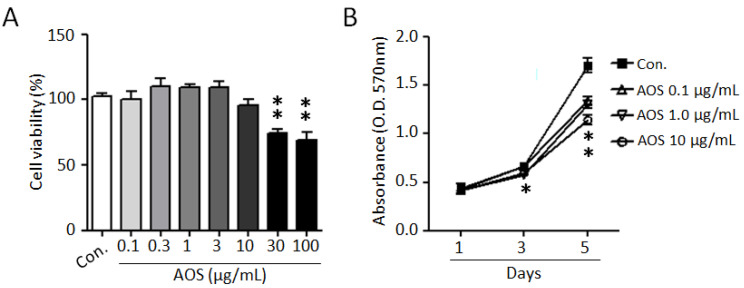
AOS inhibits the proliferation of BPH-1 cells. (**A**) BPH-1 cells were treated with different concentrations of AOS for 24 h and cytotoxicity was measured via an MTT assay. (**B**) BPH-1 cells were cultured in the absence (Con.) or the presence of AOS (0.1, 1.0, 10 μg/mL) and cell proliferation was measured after 1, 3, and 5 days. All data are shown as mean ± standard deviation of at least four independent experiments. * *p* < 0.05, and ** *p* < 0.01, compared with the control group (Con.).

To investigate the effects of AOS on the proliferation of benign prostate cells and to evaluate its cytotoxicity, we used a BPH-1 cell line. As shown in Figure 1A, AOS treatment exhibited no cytotoxicity and did not significantly affect BPH-1 cell viability up to 10 μg/mL for 24 h. Considering the elevated proliferation rates of prostate epithelial cells in benign prostatic hyperplasia, we examined the effect of AOS on the proliferation of BPH-1 cells. AOS treatment inhibited BPH-1 cell proliferation in a dose- and time-dependent manner, with the 10 μg/mL concentration having a 24% inhibitory effect after 5 days of exposure (Figure 1B). Interestingly, even at an AOS concentration of 0.1 μg/mL concentration of AOS, it was confirmed that the proliferation of BHP-1 cells was reduced by about 17% after 5 days, suggesting that AOS can inhibit the proliferation of prostate epithelial cells even at low concentrations.

**Figure 2:**
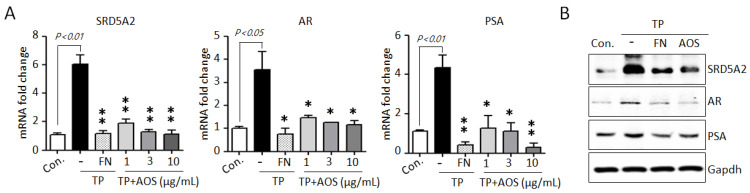
AOS inhibits the expression of 5α-reductase type 2 (SRD5A2), androgen receptor (AR), and pros-tate-specific antigen (PSA) in BPH-1 cells. (**A**) Levels of expression of SRD5A2, AR, and PSA mRNA were determined by real-time qPCR. BPH-1 cells were cultured in the absence (control; Con.) or presence of testosterone propionate (TP) 10 nM for 24 h, with or without finasteride (FN, 50 μM) or different concentrations of AOS (1, 3, and 10 μg/mL). The expression of target mRNA was normalized against that of b-actin (Actb), used as an internal control. Transcript levels are shown as fold activity relative to the control value. Data are represented as mean of three independent experiments ± standard deviation; * *p* < 0.05, and ** *p* < 0.01, compared with the control group (Con.) (**B**) Results of Western blot analysis. BPH-1 cells were cultured in the absence (Control, Con.) or presence of TP 10 nM, with 50 μM finasteride (FN) or 10 μg/mL AOS for 24 h. Glyceraldehyde-3-phosphate dehydrogenase (Gapdh) was used as an internal control. Data are representative of three independent experiments.

AR-mediated signaling, the AR-associated upstream target DHT, and the downstream target PSA are essential for cell proliferation and survival in BPH [1,2,3,12,13,14]. To investigate whether AOS treatment can suppress AR-associated pathways, we examined the expression of 5α-reductase type 2 (SRD5A2), AR, and PSA genes in BPH-1 cells in the absence or presence of TP 10 nM. Testosterone stimulation significantly induced the expression of AR-signaling associated genes, increasing the mRNA levels of SRD5A2, AR, and PSA, whereas AOS treatment inhibited the expression of these genes in a dose-dependent manner (Figure 2A). Finasteride, which specifically inhibits SRD5A2 to inhibit AR activity, results significantly in the inhibition of the expression of testosterone-induced AR-associated genes in BPH-1 cells, reducing the mRNA levels of SRD5A2, AR, and PSA (Figure 2B). Therefore, AOS can efficiently inhibit the proliferation of human prostate epithelial cells by downregulating the expression of SRD5A2, AR, and PSA without causing cytotoxicity.

### 3.2. Effect of AOS on Prostate Size in Rats with TP-Induced BPH

**Figure 3:**
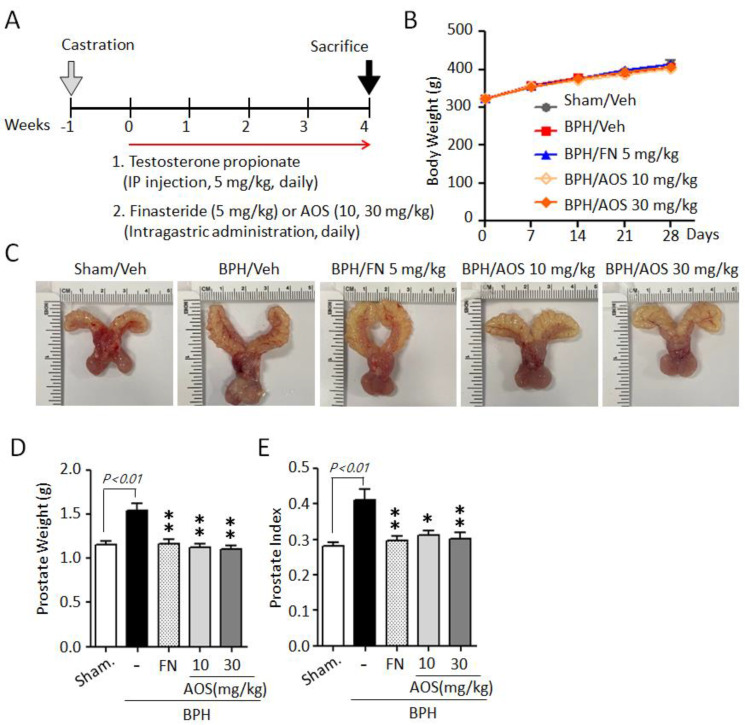
AOS administration suppresses prostate enlargement in rats with testosterone propionate (TP)-induced benign prostatic hyperplasia (BPH). (**A**) Schematic presentation of the experimental procedure. The sham-operated group (Sham/Veh), the negative control, was injected i.p. with corn oil and administered orally with the vehicle. Castrated rats were given daily i.p. injection of TP 5 mg/kg + by gavage: vehicle (BPH/Veh), 5 mg/kg finasteride (BPH/FN), or 10 or 30 mg/kg AOS (BPH/AOS) for 28 days. (**B**) Effect of AOS on body weight. (**C**) Representative images of the prostate for each experimental group. (**D**) Weight of the whole prostate. (**E**) Prostate index (the ratio of prostate weight to body weight). All data are shown as mean ± standard deviation (*n* = 5 per group). * *p* < 0.05, and ** *p* < 0.01 compared with the BPH/Veh group.

We used an orchiectomized (ORX) rat model to evaluate the in vivo effects of AOS on prostatic volume. The induction of BPH in rats is shown in Figure 3A. In the sham-operated group (Sham/Veh) or the control group, castrated rats received daily corn oil and vehicle. Castrated rats received daily TP with (BPH/FN) or without (BPH/Veh) the positive control finasteride (oral administration) or AOS (BPH/AOS) at two different doses (10 and 30 mg/kg, daily). After 28 days, no significant difference in body weight was observed (Figure 3B) between the groups. The BPH group (BPH/Veh) showed significantly increased BPH-associated parameters, such as prostate size, weight, and index, compared to the sham group (Figure 3C–E). As expected, finasteride treatment (BPH/FN) significantly reduced prostate-related parameters, the values of these parameters in the BPH/FN group being similar to those of the sham group. Interestingly, testosterone-induced BPH-associated parameters were significantly lower in AOS treatment groups (BPH/AOS) than in the control and positive control groups (Figure 3C–E). These data suggest that AOS efficiently inhibits BPH by reducing prostate enlargement in vivo.

### 3.3. Effect of AOS on the Growth of Prostate Epithelial Cells in Rats with TP-Induced BPH

**Figure 4:**
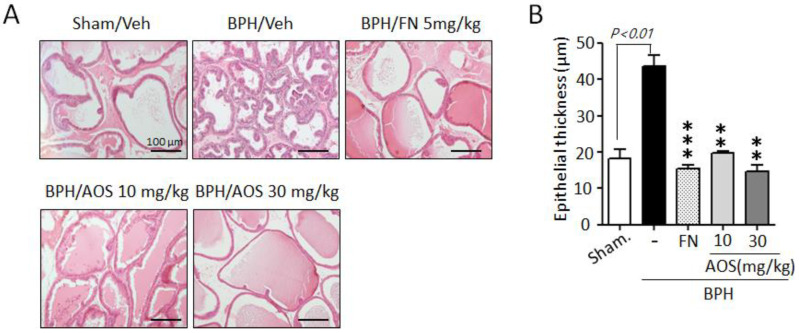
Histopathological analysis of the prostate tissues collected from rats with testosterone propionate (TP)-induced benign prostate hyperplasia (BPH). (**A**) Representative histological images using hematoxylin and eosin (H&E) staining (microscope magnification, ×100). Scale bar, 100 μm. (**B**) Quantification of the thickness of epithelial layers by ImageJ software. The graph represents the mean ± standard deviation (*n* = 3 per group); ** *p* < 0.01, and *** *p* < 0.001 compared with the BPH/Veh group. Legend: Sham/Veh, the sham-operated group used as a negative control; BPH/Veh, castrated rats receiving TP + vehicle; BPH/FN, castrated rats receiving TP + finasteride; BPH/AOS, castrated rats receiving TP + different concentrations of AOS.

To investigate the inhibitory effects of AOS on the growth and thickening of prostate epithelial cells, we performed histological staining. As shown in Figure 4A, prostate tissues in the Sham/Veh group showed acini lined by a single layer of low columnar epithelial cells linked with cuboid-shaped epithelial cells. However, the thickness of prostate tissue increased in the BPH/Veh group compared to that in the Sham/Veh group. This was a result of testosterone-induced prostate enlargement (Figure 4A,B). In contrast, the thickness of prostate epithelial cells was significantly lower in the AOS (BPH/AOS) treatment groups than in the BPH/Veh group (Figure 4A,B). Consistent with other studies, the positive control group (BPH/FN) showed obvious improvements with almost normal growth and thickness when compared to the BPH/Veh group. These results suggest that AOS can inhibit the growth of prostate cells in rats with TP-induced BPH.

**Figure 5:**
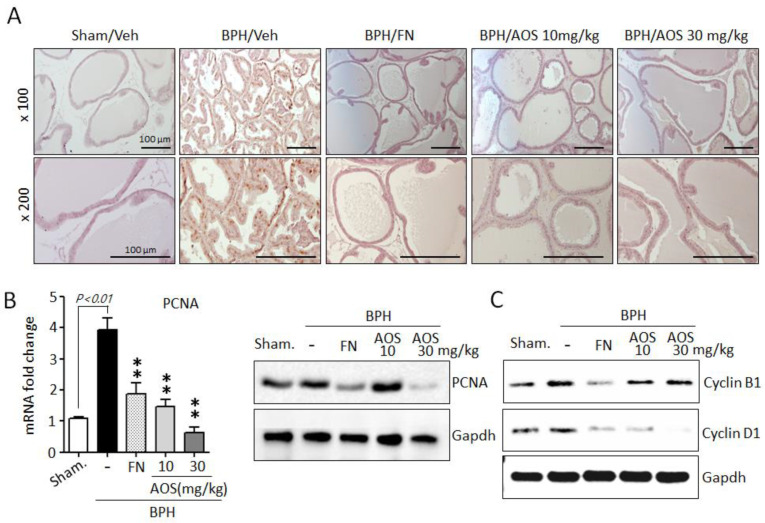
Effect of AOS on cell proliferation in the prostatic tissues of rats with testosterone propionate (TP)-induced benign prostate hyperplasia (BPH). (**A**) Representative photographs of IHC-stained prostatic tissues with anti-PCNA antibodies (magnification, ×100, and ×200). Scale bar, 100 μm. (**B**) Levels of mRNA and protein expression of PCNA in prostatic tissues were analyzed using real-time quantitative PCR and Western blot analysis. The data are presented as mean ± standard deviation (*n* = 5 per group); ** *p* < 0.01 compared with the BPH/Veh group. (**C**) Western blot analysis for assessing the protein levels of cyclin B1 and cyclin D1 in rat prostatic tissues. Glyceralde-hyde-3-phosphate dehydrogenase (Gapdh) was used as an internal control. Data are representative of three independent experiments. Legend: Sham/Veh, the sham-operated group used as a negative control; BPH/Veh, castrated rats receiving TP + vehicle; BPH/FN, castrated rats receiving TP + finasteride; BPH/AOS, castrated rats receiving TP + different concentrations of AOS.

Furthermore, using IHC and antibodies against the proliferation marker PCNA, we investigated whether AOS treatment reduced prostate cell proliferation. The expression of PCNA is shown in Figure 5A. The number of PCNA-positive cells was significantly higher in the prostate tissues of rats with TP-induced BPH than in those of the Sham/Veh group. The PCNA-positive signal was similar between the Sham/Veh, BPH/AOS and BPH/FN groups. In agreement with the histological study, the expression levels of PCNA mRNA and protein were increased in the prostate tissues of rats with TP-induced BPH compared to those in Sham/Veh rats. Testosterone-induced PCNA gene expression in prostate tissue was drastically reduced by AOS treatment (Figure 5B). Consistently, Western blot analysis demonstrated that compared to Sham/Veh rats, rats with TP-induced BPH had higher levels of expression of cyclin B1 and cyclin D1, other proliferation markers. Furthermore, treatment with AOS downregulated the expression of these markers in a dose-dependent manner (Figure 5C). Expression of cell cycle genes can be induced by AR and AR-mediated signal activation. However, upon inhibition of DHT production via finasteride treatment, expression of cell cycle genes is inhibited due to blocked AR activity. Therefore, BPH/FN group also presented lower prostatic levels of expression of cyclin B1 and cyclin D1 compared to the BPH/Veh group. These results demonstrate that AOS potently attenuates the proliferative effect of TP on prostate tissues in rats with TP-induced BPH.

### 3.4. Effect of AOS on Serum Levels of Testosterone and DHT and the Expression of AR-Associated Genes in Rats with TP-Induced BPH

**Figure 6:**
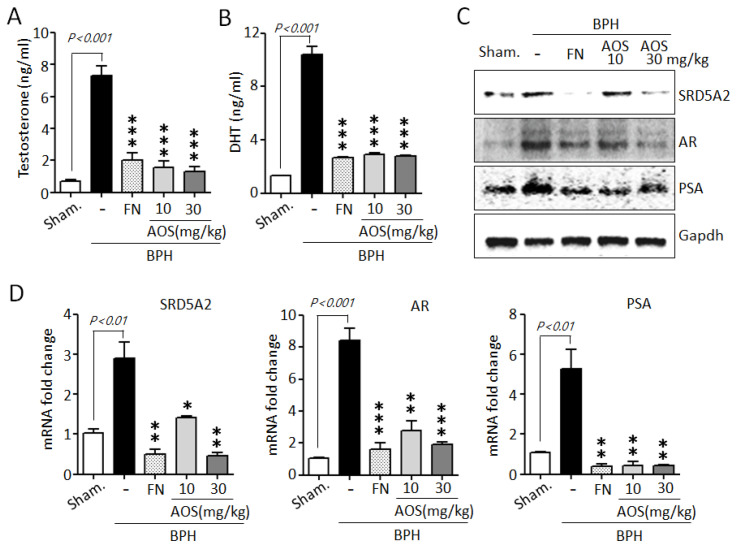
Effect of AOS on the expression of the serum levels of testosterone and dihydrotestosterone (DHT) and AR-associated genes in rats with testosterone propionate (TP)-induced benign prostate hyperplasia (BPH). Serum concentrations of testosterone (**A**) and DHT (**B**) using ELISA. (**C**) Total lysates (50 μg/lane) were extracted and visualized using Western blotting by specific antibodies. Glyceraldehyde-3-phosphate dehydrogenase (Gapdh) was used as an internal control. Data are representative of three independent experiments. (**D**) Levels of expression of SRD5A2, AR, and PSA mRNA in prostatic tissues using real-time qPCR analysis. The data are presented as mean ± standard deviation (*n* = 5 rats per group); * *p* < 0.05, ** *p* < 0.01, and *** *p* < 0.001 compared with the BPH/Veh group. Legend: Sham/Veh, the sham-operated group used as a negative control; BPH/Veh, castrated rats receiving TP + vehicle; BPH/FN, castrated rats receiving TP + finasteride; BPH/AOS, castrated rats receiving TP + different concentrations of AOS.

Testosterone is converted to DHT by the action of SRD5A2. High serum levels of testosterone and DHT and increased levels of expression of testosterone-related genes are associated with prostate growth and the pathogenesis of BPH [10,11,12,13,34,35]. As shown in Figure 6A,B, serum testosterone and DHT levels were significantly increased in the BPH/Veh group compared to those in the Sham/Veh group. However, several studies have shown that finasteride reduces serum testosterone and DHT levels. Similar to the results of earlier studies, our results show that the finasteride treatment group showed significantly reduced testosterone and DHT levels when compared to those of the BPH/Veh group. Interestingly, treatment with AOS significantly reduced the concentrations of TP-induced testosterone and DHT in the serum of rats with BPH.

To further confirm whether AOS can downregulate the expression of AR-associated genes, such as SRD5A2, AR, and PSA, in prostate tissues of rats with TP-induced BPH, we performed Western blot analysis and RT-qPCR. Consistent with the in vitro results, AOS significantly reduced the expression of SRD5A2, AR, and PSA protein and mRNA in the prostate tissues of rats with TP-induced BPH. The effect was dose-dependent (Figure 6C,D). As expected, finasteride also suppressed the levels of expression of these genes: the FN/Veh and Sham/Veh groups had similar levels of expression of SRD5A2, AR, and PSA protein and mRNA. These data suggest that AOS effectively reduced the production of DHT by inhibiting AR-associated target genes in the prostate of rats with BPH.

### 3.5. Effect of AOS on Inflammation in Rats with TP-Induced BPH

**Figure 7:**
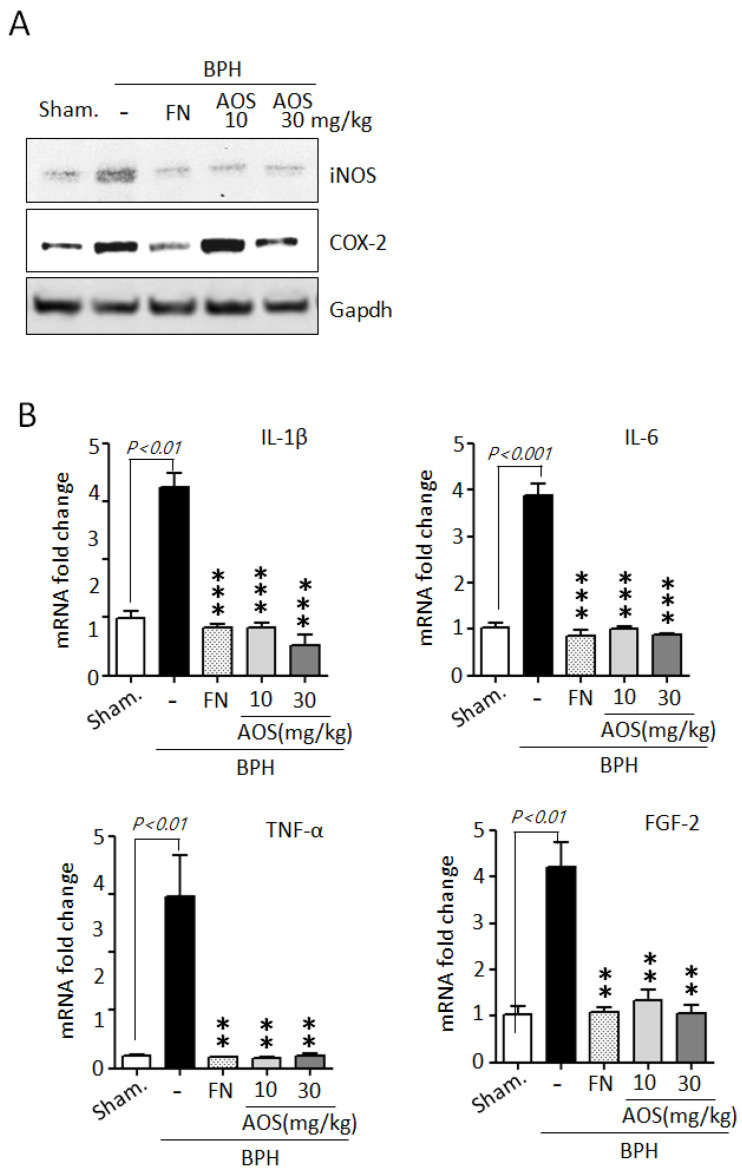
Effect of AOS on the mRNA expression of inflammation-related cytokines in prostatic tissues of rats with testosterone propionate (TP)-induced benign prostate hyperplasia (BPH). (**A**) Western blot analysis of iNOS and COX-2 protein expression in rat prostatic tissues. Glyceralde-hyde-3-phosphate dehydrogenase (Gapdh) was used as an internal control. Data are representative of three independent experiments. (**B**) The levels of mRNA expression for IL-1β, IL-6, TNF-α, and FGF2 in prostatic tissues were analyzed by real-time qPCR. The data are presented as the mean ± SD (*n* = 5 per group); ** *p* < 0.01, and *** *p* < 0.001 compared with the BPH/Veh group. Legend: Sham/Veh, the sham-operated group used as a negative control; BPH/Veh, castrated rats receiving TP + vehicle; BPH/FN, castrated rats receiving TP + finasteride; BPH/AOS, castrated rats receiving TP + different concentrations of AOS.

During BPH development and progression, prostate enlargement is also a result of inflammation mediated by several critical factors, such as enzymes and cytokines [5,6,7,8,9]. The BPH animal model indicated that inflammation in testosterone-induced prostate enlargement is particularly associated with the upregulation of iNOS and COX-2 expression. Therefore, we examined the effect of AOS on the expression of the two inducible enzymatic proteins iNOS and COX-2 in prostate tissues using Western blot analysis. As shown in Figure 7A, the protein levels of iNOS, and COX-2 were higher in the BPH/Veh group than in the Sham/Veh group. As expected, the levels of protein expression of iNOS and COX-2 declined in the Sham/Veh group after treatment with high doses of AOS or finasteride.

To further confirm whether AOS can reduce the mRNA expression of inflammatory cytokines such as IL-1β, IL-6, TNF-α, and FGF-2 in prostate tissues of rats with TP-induced BPH, we performed RT-qPCR analysis. The levels of mRNA expression of IL-1β, IL-6, TNF-α, and FGF-2 were significantly higher in prostate tissues collected from the BPH/Veh group than in those collected from the Sham/Veh group. Finasteride can attenuate the inflammatory symptoms associated with BPH by reducing the expression of inflammation-related genes. As expected, treatment with AOS or finasteride significantly decreased the levels of mRNA expression of IL-1β, IL-6, TNF-α, and FGF-2 (Figure 7B). Taken together, these results demonstrate that AOS can inhibit inflammation in the prostate of rats with BPH.

## 4. Discussion

In this study, we investigated AOS as a potential therapeutic candidate for BPH by using BPH-1 cells and a testosterone-induced rat model of BPH.

BPH is the most common benign proliferative disorder in elderly men and is a result of the overgrowth of prostatic epithelial and stromal cells. The proliferation of prostatic cells is promoted by the excessive secretion of androgens, which is a major factor in the pathogenesis of BPH [1,2,36]. In the present study, rats with TP-induced BPH showed significantly increased prostate weight and index. The histological changes in their prostatic tissue were consistent with BPH. Our study demonstrated that oral administration of AOS significantly reduced prostate weight and prostatic index, with effects similar to those of finasteride, a well-known BPH treatment agent. Moreover, we observed that AOS significantly diminished the thickness of the prostatic epithelial cell layer and stromal proliferation, using histological analysis. Consistent with the in vivo results, we found that AOS inhibited the proliferation of human BPH-1 cells in a dose- and time-dependent manner.

PCNA and cyclin D1, which act as markers of cell proliferation, play important roles in cell cycle regulation, leading to the division and duplication of prostatic stromal and epithelial cells [37,38]. PCNA is a nuclear protein that has been recognized as a histological marker of the G1/S phase of the cell cycle. Cyclin B1 and cyclin D1 are important regulators of cell cycle checkpoints, controlling G2/M and G1/S transitions, respectively. Therefore, the levels of expression of PCNA, cyclin B1, and cyclin D1 can reflect the proliferative state of prostatic cells in BPH [33,37,38,39]. In the present study, prostatic tissues in the BPH/AOS group had significantly lower levels of PCNA-positive cells than those in the BPH/Veh group, as determined by IHC, RT-qPCR, and Western blotting analyses. In addition, the levels of expression of cyclin B1 and cyclin D1 in the prostatic tissues were reduced by AOS administration via downregulation of the expression of SRD5A2 and AR. These results are consistent with those of recent studies that have demonstrated that AOS inhibits cell growth and proliferation in DU145 and PC3 prostate cancer cells [28] as well as in MG-63 bone cancer cells [40]. These results further confirm that AOS may exert preventive and therapeutic effects against prostatic hyperplasia.

BPH is a hormone-related disease, caused by an imbalance between androgenic and estrogenic effects [41]. In particular, androgens and AR-mediated signaling play pivotal roles in prostate development and growth [10,11,12,13]. In addition, testosterone and DHT play important roles in the development of male reproductive organs and are involved in prostatic hypertrophy in BPH [34,35]. The serum levels of DHT are significantly higher in men with BPH than in healthy men of similar age [42]. DHT is synthesized from circulating testosterone by the action of 5α-reductase in the prostate. Thus, 5α-reductase is a potential target for the development of inhibitors of prostatic hypertrophy. Finasteride, which specifically inhibits 5α-reductase type 2, and dutasteride, which inhibits 5α-reductase type 2 and 1, substantially decrease serum levels of DHT as well as prostate size in patients with BPH [16,17,18]. However, the long-term use of both medicines causes unwanted adverse effects [19,20]. Therefore, researchers have attempted to explore alternative treatments for BPH.

In the present study, in vivo, and in vitro results indicated that AOS attenuated prostatic hypertrophy and pathological changes by inhibiting the proliferation of prostate epithelial cells, reducing the expression of testosterone-induced target genes SRD5A2, AR, and PSA, and decreasing the serum levels of testosterone and DHT. The results were comparable to those of finasteride, used as a positive control. Furthermore, we confirmed that AOS reduced the expression of inflammatory cytokines and enzymes in the prostate of rats with BPH. These results suggest that AOS effectively inhibits cell proliferation in BPH and may be a viable alternative to 5α-reductase inhibitors for treating this pathology.

Gene expression profiling performed on cell cultures and tissues from patients with BPH has provided insights into the potential role of inflammatory cytokines and enzymes as well as growth factors in the pathogenesis of BPH. A causal relationship exists between prostatic inflammation and the development of BPH [5,6,43]. BPH tissues and prostate cancer cells express toll-like receptor 4 (TLR4) and are characterized by increased immune-mediated inflammatory processes and secretion of cytokines, such as IL-1β, IL-6, IL-8, TNF-α, and COX-2 [9,44,45]. In addition, lipopolysaccharide stimulation can induce the production of nitric oxide in prostatic epithelial cells [46]. The inflammation-related genes suggest a key role of innate immunity in the development of chronic inflammation, tissue remodeling, and enlargement of the prostate in BPH. Additionally, many growth factors have been investigated as inducers of chronic inflammation and abnormal stromal and epithelial cell growth in BPH [47]. Moreover, the expression of FGF-2 and its receptor FGFR1 is upregulated in BPH tissue compared to that in normal tissue [48,49], suggesting that stimulation of the FGF-2 signaling pathway is associated with BPH. Therefore, many research groups have investigated compounds that inhibit the release of inflammatory cytokines as potentially effective therapeutic agents for BPH. In the present study, the expression of the inflammation-related genes encoding iNOS and COX-2 and inflammatory cytokines IL-1β, IL-6, and TNF-α was significantly higher in the prostatic tissue of rats with TP-induced BPH than in the tissue of sham-operated rats. However, treatment with AOS downregulated the expression of these genes in a dose-dependent manner. Moreover, in the prostatic tissue of rats, the TP-induced increase in the mRNA expression of FGF-2 was significantly inhibited by the oral administration of AOS. In the current study, we demonstrated that AOS is an effective anti-inflammatory agent, which can reduce prostatic hyperplasia, suggesting that it may be an effective therapeutic agent for BPH.

Through several studies, it has been suggested that interactions between host cells and gut microbiota or their metabolic products may affect prostate enlargement. Changes in gut microbiota populations can promote inflammatory properties in the prostate through the production of proinflammatory cytokines such as IL-17, IL-23, TNF-alpha, and IFN-gamma [50]. In addition, patients with BPH had decreased numbers of Firmicutes and significantly increased numbers of Bacteroidales among their gut microbiota; additionally, short-chain fatty acids (SCFA) levels increased from intestinal bacteria, promoting prostate enlargement by activating the IGF-1 signaling pathway [51]. Moreover, prostate enlargement is associated with inflammation, changes in hormone and cytokine levels, and metabolic syndrome [52]. AOS increases beneficial gut bacteria such as Bacteroidales and Lactobacillaceae, while it decreases harmful bacteria such as Desulfovibrionaceae in the feces of AOS-dosed mice [53]. AOS treatment could significantly enrich several SCFA-producing bacteria that are mainly involved in fiber degradation and butyrate production. SCFA-producing bacteria can provide energy sources for enterocytes, protect intestinal mucosa integrity, regulate immunity, and reduce inflammation [54]. Also, AOS administration has been shown to exert anti-inflammatory effects through the activation of the AMPK signaling pathway and modifying gut microbiota composition. It has been suggested that AOS administration can improve intestinal health by enriching beneficial bacteria and inhibiting the proliferation of detrimental bacteria, thus reducing prostatic hyperplasia.

Over the last decades, AOS as a biocompatible, nonimmunogenic, and nontoxic material, has increasingly drawn attention as an attractive compound in the biological and pharmaceutical areas due to its versatile biological activities. Through recent technological advancements, AOS has been extensively studied for a wide range of applications, including antimicrobial, antiviral therapy, diabetes, and neurodegenerative disease treatment [55,56,57,58]. In the present study, we provide the first demonstration that AOS may be effective at treating prostatic hyperplasia. However, further studies are necessary to identify the molecular mechanisms for the efficacy of AOS in prostatic hyperplasia. Although we have not yet tested the anti-BPH effect of AOS on developed prostatic hyperplasia, it might be interesting to investigate in the future.

## 5. Conclusions

In conclusion, our results demonstrated that AOS decreased prostate weight and the expression of SRD5A2, AR, and PSA in rats with testosterone-induced BPH, with minimal effects on body weight. AOS demonstrated anti-proliferative and anti-inflammatory effects. Although further clinical trials and safety studies in humans are required, our findings suggest that AOS could be potentially useful as a dietary supplement for the treatment of BPH, effectively slowing its progression.

## Figures and Tables

**Table 1:** Sequences of primers used for real-time qPCR.

Target Gene		Sequences
SRD5A2 (Human) AR (Human) PSA (Human) β-actin (Human) SRD5A2 (Rat) AR (Rat) PSA (Rat) PCNA (Rat) IL-1β (Rat) IL-6 (Rat) TNF-α (Rat) FGF-2 (Rat) β-actin (Rat)	Sense Antisense Sense Antisense Sense Antisense Sense Antisense Sense Antisense Sense Antisense Sense Antisense Sense Antisense Sense Antisense Sense Antisense Sense Antisense Sense Antisense Sense Antisense	TGAATACCCTGATGGGTGGT GGAAATTGGCTCCAGAAACA CTCACCAAGCTCCTGGACTC CAGGCAGAAGACATCTGAAAG GCAGCATTGAACCAGAGGAG AGAACTGGGGAGGCTTGAGT GATGAGATTGGCATGGCTT CACCTTCACCGTTCCAGTTT ATTTGTGTGGCAGAGAGAGG TTGATTGACTGCCTGGATGG GGGTGACTTCTCTGCCTCTG CCATCCAAGGTCCCATTTC GGGGGCAAAGATATATGCAA GCACACCATCACAAATGAGG CAATTTCTAGCAACGCCTAAGAT AAGAGGAAGCTGTGTCCATAGAG TCCTCTGTGACTCGTGGGAT TCAGACAGCACGAGGCATTT AGAGACTTCCAGCCAGTTGC AGCCTCCGACTTGTGAAGTG TCGTCTACTCCTCAGAGCCC ACTTCAGCGTCTCGTGTGTT GAACCGGTACCTGGCTATGA CCGTTTTGGATCCGAGTTTA CGTGAAAAGATGACCCAGAT ACCCTCATAGATGGGCACA

## Data Availability

The data presented in this study are available from the corresponding author upon reasonable request.

## Abbreviations

BPH	Benign prostatic hyperplasia
AOS	Alginate oligosaccharide
AR	Androgen receptor
SRD5A2	5α-reductase type 2
PSA	Prostate-specific antigen
LUTS	Lower urinary tract symptoms
DHT	Dihydrotestosterone
PCNA	Proliferation cell nuclear antigen
iNOS	Inducible nitric oxide synthase
COX-2	Cyclooxygenase-2
Gapdh	Glyceraldehyde-3-phosphate dehydrogenase
TNF-α	Tumor necrosis factor-α
FGF-2	Fibroblast growth factor-2
